# Supplementary material for: Pharmacodynamic and Toxicity Studies of 6-Isopropyldithio-2′-guanosine Analogs in Acute T-Lymphoblastic Leukemia
Source: Cancers (Basel). 2024 Apr 23;16(9):1614. doi: 10.3390/cancers16091614 (PMC11083707; doi:10.3390/cancers16091614)
Supplement: Supplementary file 1 [file cancers-16-01614-s001.zip › cancers-2907012-supplementary.pdf]

Supplementary Materials:.

Table S1: Routine blood test results of acute toxicity test in mice.

	WBC	RBC	Hb	MCV	PLT	LYMPH	MXD
Blank	7.44±2.88	9.39±0.37	170±1.41	52.55±1.06	565.5±0.71	6.29±2.13	0.3±0.17
307 mg/kg	6.1±2.73	7.81±0.13**	148.5±4.95	57.9±0.71	580±76.37	3.05±0.91	0.34±0.24
350 mg/kg	4.61±2.23	7.64±0.06**	151±5.66	58.7±2.26	579±100.41	3.01±1.11	0.22±0.13
380 mg/kg	3.62±0.54	7.33±0.02***	134.5±3.54*	56.85±2.62	514±125.87	2±0.4	0.17±0.1
414 mg/kg	6.21±3.03	8.54±0.04*	159±12.73	58.95±4.31	512.5±130.81**	2.76±1.37	0.4±0.18
	**DLC**	**LY%**	**MID%**	**DLC%**	**P-LCR**	**B-PLT**	**HCT**
Blank	0.85±0.58	85.4±4.38	3.9±0.71	10.7±3.68	23.8±4.81	134.5±27.58	49.3±0.99
307 mg/kg	2.72±1.58	51.75±8.13*	5.25±1.63	43±6.51**	14.75±4.31	84±14.14	45.2±1.27
350 mg/kg	1.39±1	67.25±8.56	4.6±0.57	28.15±7.99	26.2±8.91	156±77.78	44.85±1.34
380 mg/kg	1.45±0.04	55±2.83*	4.65±2.05	40.35±4.88*	14.8±5.66	72.5±10.61	41.65±1.77
414 mg/kg	3.05±1.48	44.45±0.35*	6.4±0.28	49.15±0.07**	26.85±7.71	132.5±4.95	50.3±3.54
	**MCHC**	**RDW**	**RDW-SD**	**MCH**	**MPV**	**PCT**	**PDW**
Blank	345±9.9	11.85±0.49	61.6±1.56	18.15±0.92	9.3±0.14	0.53±0.01	19.3±0.14
307 mg/kg	328.5±2.12	13.65±2.33	70.4±1.41	19±0.28	9.85±0.07	0.57±0.07	20±0.42
350 mg/kg	336.5±2.12	12.95±1.91	70.85±6.72	19.75±0.92	9.85±0.21	0.57±0.11	20.1±0.85
380 mg/kg	323±5.66	12.55±0.07*	65.8±6.79	18.35±0.49	9.55±0.07	0.49±0.12	20.6±0.71
414 mg/kg	316±2.83	11.65±0.07*	72.95±15.63	18.6±1.56	10±0.57	0.51±0.1**	18.5±1.7**

The mice in the 414 mg/kg group showed a decrease in erythrocyte content, a decrease in the percentage of lymphocytes and an increase in the percentage of granulocytes. *$p < 0.05$,**$p < 0.01$,*** $p < 0.001$,**** $p < 0.0001$.

Table S2: The results of biochemical indicators of acute toxicity test in mice.

	ALT	AST	TBIL	ALB	CREA	BUN	TG
Blank	4.9±2.69	6.65±3.46	2.35±1.34	2.6±1.56	2.95±1.06	0.56±0.34	0.07±0.05
307 mg/kg	6.35±2.62	5.65±1.34	3.2±1.13	3.15±0.21	3.5±1.41	0.57±0.22	0.07±0.02

350 mg/kg	4.75±0.78	7.3±3.82	3.65±0.21	3.45±0.07	5.25±1.48	0.67±0.05	0.08±0.02
380 mg/kg	4.85±1.48	5.9±0.42	3.25±0.21	3.5±0.57	2.8±0.85	0.61±0.06	0.16±0.04
414 mg/kg	4.65±1.91	9.05±0.07	3.55±0.21	3.35±0.35	3.7±2.4	0.58±0.16	0.09±0.01

	TC	ALP	GLU	K	CL	CK
Blank	0.07±0.04	11±7.07	0.45±0.18	2.27±0.09	37.5±21.78	29.5±20.51
307 mg/kg	0.1±0.04	12±2.83	1.24±0.76	2.42±0.06	50.1±10.47	27±8.49
350 mg/kg	0.18±0.06	13±8.49	1.85±0.97	1.69±1.29	46.6±2.4	27±18.38
380 mg/kg	0.14±0.05	13.5±2.12	0.89±0.06	2.46±0.08	57.1±0.14	30±4.24
414 mg/kg	0.08±0.01	9.5±2.12	0.44±0.59	2.43±0.02	56.4±1.84	19±0

No abnormal changes in biochemical indices in serum of mice. * $p < 0.05$,**$p < 0.01$,*** $p < 0.001$,**** $p < 0.0001$.

Table S3: Routine blood test results of acute toxicity test in rat.

	WBC	RBC	Hb	MCV	PLT	LYMPH	MXD
Blank	8.99±2.44	6.65±0.24	182.75±58.27	64.95±1.82	507.5±158.55	7.32±1.6	0.28±0.09
0.9% NaCl	7.22±0.99	7.39±1.5	159.83±27.24	63.3±0.73	415.83±129.92	6.03±1.05	0.24±0.04
1 mg/kg	7.03±2.23	14.04±22.15	162.9±31.29	65.67±3.24	553.8±64.37	5.81±2.05	0.23±0.08
2 mg/kg	3.76±2.35***	5.08±1.55	114.33±34.58*	62.8±2.21	330±233.71	3.09±1.67***	0.11±0.11*
10 mg/kg	14.45±36.82****	6.95±1.42	157.33±33.74	63.19±2.18	203.56±44.74**	10.32±24.98****	0.59±1.64***

	DLC	LY%	MID%	DLC%	P-LCR	B-PLT	HCT
Blank	1.4±0.84	82.45±6.32	3.05±0.42	14.5±6.06	38.83±6.77	190.25±40.75	43.15±1.08
0.9% NaCl	0.95±0.19	83.15±4.39	3.32±0.42	13.53±4.12	40.67±3.89	165±40.14	46.77±9.57
1 mg/kg	0.99±0.6	82.41±9.3	3.32±0.61	14.27±8.72	36.55±3.29	202.7±31.31	46.91±9.41
2 mg/kg	0.57±0.6	87.61±10.91	2.07±1.65	10.32±9.29	39.79±5.97	120.89±72.91	32.08±10.26
10 mg/kg	3.6±10.18**	90.46±11.81	1.27±1.37**	8.28±10.57	45.79±22.41	96.89±62.89	43.92±9.29

	MCHC	RDW	RDW-SD	MCH	MPV	PCT	PDW
Blank	425.5±145.7	11.6±0.74	64.13±5.79	27.5±8.81	10.65±0.13	0.54±0.17	18.03±1
0.9% NaCl	344.17±14.85	12.03±0.87	65.17±4.2	21.77±1.03	10.45±0.14	0.43±0.13	17.07±0.85

1 mg/kg	347.8±8.38	12.15±1.39	66.84±8.4	22.86±1.14	10.67±0.33	0.59±0.08	18.06±0.88
2 mg/kg	359.11±12.84	10.42±1.54	60.47±6.33	22.51±0.54	10.33±0.3	0.35±0.25	18.21±1.22
10 mg/kg	358.56±22.92	11.24±1.48	61.6±6.02	22.68±1.94	10.48±0.26	0.21±0.05**	15.82±0.64**

Rats in the 10 mg/kg group showed significant changes in several blood indices. * $p < 0.05$,**$p < 0.01$,*** $p < 0.001$,**** $p < 0.0001$.

Table S4: The results of biochemical indicators of acute toxicity test in rat.

	ALT	AST	TBIL	ALB	CREA	BUN	TG
Blank	37.35±3.72	112.45±15.58	3.43±1.75	19.88±2.82	11.18±4.7	1.35±0.49	0.41±0.16
0.9% NaCl	33.37±4.49	112.65±20.81	3.33±0.65	20.35±3.71	8.38±3.1	1.54±0.37	0.21±0.09
1 mg/kg	38.04±10.62	135.82±32.39	3.68±0.93	21.11±2.02	11.89±3.74	1.82±0.4	0.38±0.2
2 mg/kg	27.06±7.24	86.44±29.98	3.53±0.94	19.19±3.94	9.12±4.59	1.72±0.43	0.35±0.28
10 mg/kg	19.54±12.18*	74.52±30.35	3.22±0.91	18.74±2.47	11.08±4.5	2.47±0.84*	0.24±0.04

	TC	ALP	GLU	K	CL	CK
Blank	0.81±0.22	116.75±14.66	2.57±0.86	2.62±0.48	51.83±1.71	846.5±296.78
0.9% NaCl	0.63±0.17	97.83±40.29	2.52±0.59	2±0.26	53.63±7.08	624±185.35
1 mg/kg	0.6±0.22	96.2±26.72	2.84±0.49	2.27±0.35	53.46±3.19	857.8±280.32
2 mg/kg	0.69±0.21	105.56±35.36	3.54±0.72	2.52±0.42	55.79±4.37	507.11±351.9
10 mg/kg	0.45±0.17*	37.44±14.23***	2.87±0.44	2.06±0.43	56.2±2.92	187.89±57.27**

At 10 mg/kg, significant changes in several serum biochemical indices were observed in rats. *$p < 0.05$,**$p < 0.01$,*** $p < 0.001$,**** $p < 0.0001$.

Figure S1:Western blot analysis was conducted without any image cropping.

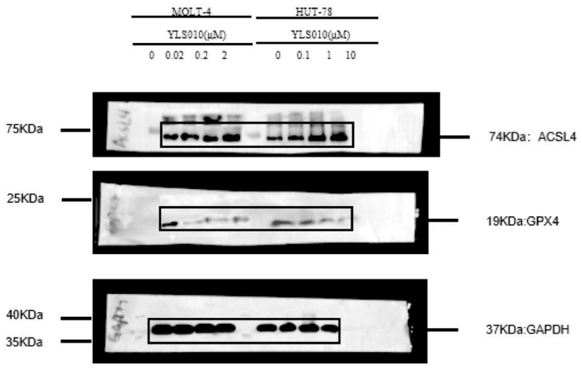
MOLT-4
HUT-78
YLS010(μM)
YLS010(μM)
0 0.02 0.2 2
0 0.1 1 10
75KDa
74KDa: ACSL4
25KDa
19KDa:GPX4
40KDa
35KDa
37KDa:GAPDH

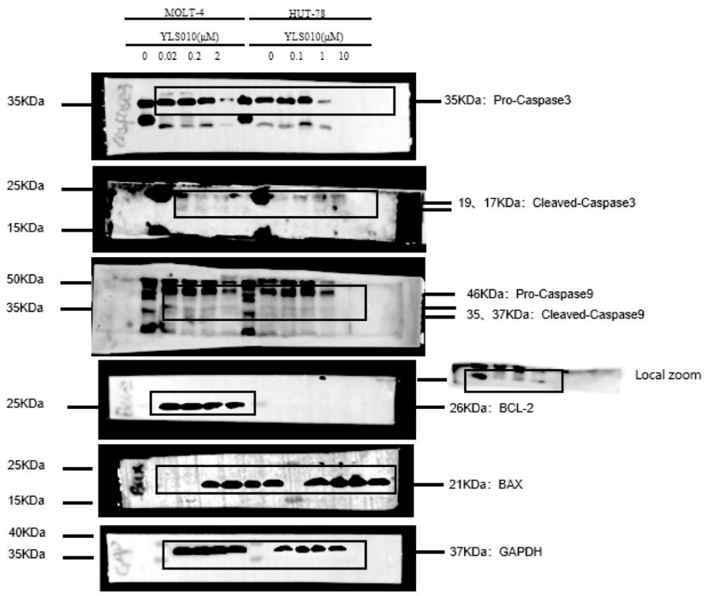
MOLT-4
HUT-78
YLS010(μM)
YLS010(μM)
0 0.02 0.2 2
0 0.1 1 10
35KDa
35KDa: Pro-Caspase3
25KDa
15KDa
19、17KDa: Cleaved-Caspase3
50KDa
35KDa
46KDa: Pro-Caspase9
35、37KDa: Cleaved-Caspase9
Local zoom
25KDa
26KDa: BCL-2
25KDa
15KDa
21KDa: BAX
40KDa
35KDa
37KDa: GAPDH
